# Supplementary material for: Chromothripsis during telomere crisis is independent of NHEJ, and consistent with a replicative origin
Source: Genome Res. 2019 May;29(5):737–49. doi: 10.1101/gr.240705.118 (PMC6499312; doi:10.1101/gr.240705.118)
Supplement: Supplemental Material [file supp_gr.240705.118_Supplemental_file_1.zip › contigs/annotated_contigs/DB92/contig.5.DB92_length_496_mean_cov_5.31048387097.docx]

**DB92_length_496_mean_cov_5.31048387097**

```
AAAGAAAAAAAACTCTCAGCCAATAATCACGTATCCAACAAAACTGTACTGCAAAAGAAGTAAAGGA|ATGCTAGACCATTAACACTAG
 >chr3:59832542-59832609 - E=1e-25
TTAGAACTAGATCTGCCCTTCAAGAAATG|GT|GAGAATAACGGATTTTTAAAACATATTGGTCTAAAAATCAATATTATTATAACTTT
 >chr3:59832466-59832495 - E=8e-06  >chr3:59832205-59832303 - E=2e-44 p=4e-03
GGTTTATAACTCCATGTTTTGTTTTCTATATAATTTAAGAGA|TT|ATGATGTTAAGAAAAATAACCTTTCCTTTTTTTCTTTGCTTTC
                                             >chr3:60922465-60922557 - E=9e-44 p=5e-03
TTTGTATTAAAAAATGAAAATTATCCTCTTTTCCCTTTGAGTTCAT|GCT|CTAGAAGAAGAGGATTCATTTTCTACATAGGCAATAAT
                                               >chr3:60922153-60922341 - E=2e-95
GTATGTTATAAAAAATCAAAAGATAGTCACATGGAAAGGGGAAAAATGAATGTAGATCAATAATTTAACTCTGACCTAAGTTCTATAAC

ATGGCTCATAATTTTTCAATGAATAGACCCTTAACATTTTACATATACAGCGTCAGTT
```
